# The Oral Health Status of Adults With Special Healthcare Needs: A Hospital-Based Study and Comparative Analysis With National Data

**DOI:** 10.7759/cureus.88796

**Published:** 2025-07-26

**Authors:** Mohamad Noor Sairi, Nor Azlida Mohd Nor, Ainol Haniza Kherul Anuwar, Maryani Mohamed Rohani, Aisyah Ahmad Fisal

**Affiliations:** 1 Department of Community Oral Health and Clinical Prevention, Faculty of Dentistry, Universiti Malaya, Kuala Lumpur, MYS; 2 Department of Paediatric Dentistry and Orthodontics, Faculty of Dentistry, Universiti Malaya, Kuala Lumpur, MYS

**Keywords:** disability, periodontal status, special health care dentistry, special healthcare needs (shcn), dental caries

## Abstract

Background and objective

There is a growing need to address the oral health of individuals with special healthcare needs (SHCN). However, research data on this population is often limited, particularly among adults and the elderly. Hence, this study aimed to assess the oral health status of adult SHCN patients attending a Malaysian dental hospital, identify associated factors, and compare their disease burden with the national prevalence.

Methodology

This was a cross-sectional study involving retrospective data analysis of adult SHCN patients who received treatment at a Malaysian dental hospital between January 2021 and December 2023. Data on sociodemographic characteristics, medical conditions, disability types, complexity level, dental caries status, and periodontal health were collected. Dental caries and periodontal disease were measured using the International Caries Detection and Assessment System (ICDAS) and Basic Periodontal Examination (BPE) indices, respectively. Additionally, oral health data from the general adult population were extracted from the Malaysian National Oral Health Adult Survey 2020. The data were analyzed using descriptive and multivariate analyses.

Results

A total of 513 adult patients were included in the analysis. More than half (n = 271, 52.8%) were male, with a mean age of 51.04 years (SD: ±21.36). The prevalence of dental caries was significantly lower in the SHCN group compared to the national average [n = 370, (72.1%) vs. n = 14,241, (85.1%)]. However, the SHCN group had fewer remaining natural teeth than the general population, with a mean of 22.6 and 24.4, respectively. Although the general population had a higher overall prevalence of unhealthy periodontium [n = 15,814 (94.5%) vs. n = 418 (81.5%)], the SHCN group exhibited a significantly higher prevalence of periodontal pockets [n = 6,392 (38.2%) vs. n = 244 (47.6%)]. Age remained a significant predictor of dental caries in the regression model. Periodontal disease was more common among older adults and those with multiple medical conditions or disabilities. Additionally, patients with a moderate complexity score had a significantly higher risk of developing periodontal pockets (BPE score ≥3) (p = 0.032).

Conclusions

The prevalence of oral disease among adults with SHCN was generally comparable to the national average, with a notably higher burden of advanced periodontal disease and lower overall tooth retention. Age, multiple disabilities, and higher complexity levels were associated with poor oral health status in this population. These findings underscore the need to strengthen and expand oral healthcare delivery through multi-sectoral collaboration for this vulnerable group.

## Introduction

Approximately 16% of the global population, or about 1.3 billion people, live with some form of disability [1]. In response to this, the World Health Organization (WHO) emphasizes the importance of developing inclusive and equitable healthcare systems, particularly for individuals with complex needs, such as persons with special healthcare needs (SHCN). This vulnerable group includes individuals who have chronic physical, developmental, behavioral, or emotional conditions that require healthcare services beyond what is typically needed by the general population [2-3].

Despite the growing numbers, access to quality oral healthcare remains a significant concern for this vulnerable population. A systematic review has shown that persons with SHCN commonly experience limited access to dental services, poorer oral health status, and low oral health awareness [4]. This issue is particularly pressing as many countries, including Malaysia, are transitioning into super-aged nation status [5]. As populations age, there is a corresponding rise in disability and complex healthcare needs, which significantly increases the demand for accessible and specialized oral healthcare services [4,6]. It is, therefore, crucial to address the oral health needs of the aging and SHCN populations to help reduce health disparities.

Malaysia is among the countries with the most heavily subsidized public healthcare systems, and initiatives have been undertaken to improve access to specialized dental care for populations with SHCN. National survey data indicate that 11.1% of Malaysians aged 18 years and above have some form of disability [7], and the proportion of the population aged 60 years and above has steadily increased from 7.9% in 2010 to 11.1% in 2020 [5]. Additionally, the number of adult patients with SHCN attending public Special Care Dentistry (SCD) specialist clinics has increased since 2017, with a 36.4% rise in attendance recorded in 2022 compared to 2021 [8]. The increase in service utilization reflects some progress in accessibility, but SHCN continue to encounter other barriers, including inadequate healthcare infrastructure, a shortage of trained personnel, and communication difficulties [3]. These factors contribute to an increase in the burden of oral diseases, negatively affecting the quality of life for both patients and their caregivers.

Therefore, more local data are needed to aid service planning and resource allocation, especially given Malaysia’s increasing population of individuals with SHCN and its aging society. Most existing local research concentrates on children and adolescents with SHCN [2,9]. Thus, this study's objectives were to 1) evaluate the oral health status of adult SHCN patients at a Malaysian dental hospital; 2) identify factors associated with their oral health outcomes; and 3) compare their disease burden with national prevalence data. The null hypothesis proposed that there was no significant difference in oral health status between adult SHCN patients at a Malaysian dental hospital and the general adult population based on national data.

## Materials and methods

Ethical approval and standard of reporting

This study was designed and conducted in accordance with the Declaration of Helsinki. The study protocol was approved by the Medical Ethics Committee, Faculty of Dentistry, Universiti Malaya (FODUM), Malaysia (Reference No.: DF CO2402/0006 (P)). The reporting of this study adhered to the STROBE (Strengthening the Reporting of Observational Studies in Epidemiology) guidelines for cross-sectional studies.

Study design, samples, and data collection

This was a cross-sectional study involving retrospective data analysis of patients with SHCN at a public university dental hospital in Kuala Lumpur, Malaysia. These patients were treated by SCD specialists and postgraduate residents at the hospital. This hospital functions as a referral centre for SCD and thus provides a valuable context for comparison with national oral health data. A universal sampling approach was used, including all SHCN patients who visited the SCD clinics at the FODUM between January 2021 and December 2023. January 2021 was selected as the starting point to reflect the post-COVID-19 clinic operation procedures. The data collection period was from March to July 2024. The sample size calculation was based on the formula for estimating a single proportion, using caries prevalence data (45%) from a previous study by Affandi et al. (2023) [2], with a precision of 5% and a power of 0.80. The minimum required sample size was 297. As the number of available patient records exceeded the minimum requirement, all records were included to ensure sufficient power for the analysis.

Relevant data were extracted from the patient digital records system. Only complete records of adult patients (aged 16 years and above) were included in the analysis. The age limit of 16 years was determined according to the eligibility criteria for SCD services within the Malaysian population [8]. Records were excluded if they contained incomplete data (i.e., missing clinical data such as caries assessment/full mouth charting, periodontal status, or sociodemographic information) or if patients received care solely through the domiciliary dental services or outreach programmes without ever attending the SCD clinic.

A standardized data extraction form was used during the data collection process. A Microsoft Excel data extraction form was developed and used to systematically collect information from patient records. The data extraction form was piloted using 20 patient records, and necessary amendments were made to the form before the main data collection. The following information was collected: sociodemographic information, types of disability and medical conditions, level of complexity, clinical diagnoses, as well as oral health status on caries and periodontal diseases. Disability status (yes/no) was determined based on the possession of a national disability registration card. Patient complexity was based on British Dental Association (BDA) case mix classification (0 = standard complexity, 1-9 = some complexity, 10-19 = moderate complexity, 20-29 = severe complexity, ≥30 = extreme complexity [10]. The clinical data were extracted based on their first dental visit to the SCD clinic.

Dental caries status was measured according to the International Caries Detection and Assessment System (ICDAS) score as charted by the clinicians and subsequently recoded into three categories ( D_0 _= healthy, D_1-3 _= early/enamel caries, D_4-6_ = dentine caries) [11]. These scores were further converted into the caries experience DMFT (Decayed, Missing, Filled, Teeth) score, with D_3-6_ representing cavitated caries lesions [11]. The periodontal health status of the patients was assessed through the clinicians’ notes on the condition of the gums and periodontal pocket depth, as well as Basic Periodontal Examination (BPE) scores [12]. Each patient’s periodontal health status was categorized based on the most severe condition observed in the mouth. BPE scores were classified as follows: healthy (BPE 0), gingivitis (BPE 1-2, characterized by bleeding on probing and the presence of calculus), and periodontitis (BPE 3-4, indicating the presence of periodontal pockets). The data were organized and analyzed in Microsoft Excel by the investigator (MNS) and cross-checked by two other researchers (NAMN, AHKA) before being transferred to SPSS Statistics software.

To test the null hypothesis, the oral health status from this study was compared with that of the general adult population in Malaysia, as reported in the National Oral Health Survey of Adults (2020) [13]. The national survey involved Malaysian adults aged 15 years and older.

Data analysis

Data were analyzed with the SPSS Statistics software, version 29.0 (IBM Corp., Armonk, NY), using both descriptive and multivariate statistics. Categorical variables were presented using frequencies and percentages, while continuous variables were summarized using means and standard deviations (SD). Each variable in the data set was subjected to the Kolmogorov-Smirnov test to evaluate its distribution for normality. For comparisons between two groups, independent t-tests (for continuous data) and chi-square tests (for categorical data) were employed. Additionally, binary logistic regressions were performed to identify significant predictors associated with the risk of having dental caries and periodontal disease. Effect size estimates were reported as adjusted odds ratios (AOR) derived from binary logistic regression models. Each AOR was accompanied by a 95% confidence interval (CI) to indicate the precision of the estimate.

## Results

A total of 513 SHCN patient records fulfilled the inclusion criteria for further analysis. Overall, the study population comprised more males (n = 271, 52.8%) than females (n = 242, 47.2%), with a mean age of 51.0 (SD: ±21.36). In terms of ethnicity, most participants were Chinese (n = 232, 45.2%), followed by Malay (n = 202, 39.4%) and Indian (n = 79, 15.4%) (Table 1). More than one-third of the study samples were elderly patients aged 61-73 years. The proportion of patients with a nationally registered disability card (n = 342, 66.7%) was significantly greater than the proportion without the card (p<0.001). The classification of disabilities, based on local guidelines, encompassed developmental, neurological, and medically complex conditions, as well as social impairments, psychiatric illnesses, sensory, and physical disabilities [2,8]. More than half of the patients had a combination of two or more disabilities (Table 1).

**Table 1:** Demographic characteristics of SHCN patients attending SCD clinics from January 2021 to December 2023 (n = 513) ^a^Chi-square test.^ *^The level of significance for this analysis was set at 0.05 BDA: British Dental Association; SCD: Special Care Dentistry; SHCN: special healthcare needs

Variables	Frequency	%	P-value^a^	Chi-square value
Gender				
Male	271	52.8	0.20	1.6
Female	242	47.2		
Age group, years				
16–20	36	7.0	<0.001^*^	92.7
21–30	95	18.5		
31–40	55	10.7		
41–50	54	10.6		
51–60	76	14.8		
61–73	197	38.4		
Ethnicity				
Malay	202	39.4	<0.001^*^	76.9
Chinese	232	45.2		
Indian	79	15.4		
Patient with a nationally registered disability card				
Yes	342	66.7	<0.001^*^	57.0
No	171	33.3		
Number of disabilities				
1 type of disability	216	42.1	<0.001^*^	25.4
2 types disabilities	123	24.0		
≥3 types of disabilities	174	33.9		
Total number of medical conditions				
None	45	8.8	<0.001^*^	422.8
1 medical condition	183	35.7		
2 medical conditions	154	30.0		
≥3 medical conditions	131	25.5		
Patient complexity (BDA case-mix score)				
Standard complexity	33	6.4	<0.001^*^	171.0
Some complexity	152	29.6		
Moderate complexity	183	35.7		
Severe complexity	104	20.3		
Extreme complexity	41	8.0		

The association between dental caries and patient demographic characteristics is shown in Table 2. Age and number of medical conditions were significantly associated with dental caries. Overall, the prevalence of dentine caries (n = 360, 70.2%) was higher than that of enamel caries (n = 74, 29.8%). Males exhibited a higher prevalence of dentine caries (181, 35.3%) compared to females. However, the difference was not statistically significant. In terms of age, older age groups, particularly those aged 61 years and above, showed significantly higher prevalence of both enamel (n = 38, 7.4%) and dentine caries (n = 153, 29.8%). Patients with a moderate level of complexity had a significantly higher prevalence of dentine caries (n = 137, 6.7%) compared to those in other complexity levels (p<0.001).

**Table 2:** The association between dental caries at the baseline appointment and patients’ demographic characteristics (n = 513) ^a^Chi square test. ^*^The level of significance for this analysis was set at 0.05 D1-3: caries at the enamel level. D4-6: caries at the dentine level BDA: British Dental Association

Variables	Frequency, n (%)					
	Caries-free	Enamel caries	Dentine caries	Total	P-value^a^	Chi-square value
Gender						
Male	45 (8.8)	45 (8.8)	181 (35.3)	271 (52.8)	0.185	3.4
Female	34 (6.6)	29 (5.7)	179 (34.9)	242 (47.2)		
Age group, years						
16–20	26 (5.1)	2 (0.4)	8 (1.6)	36 (7.0)	<0.001*	118.3
21–30	31 (6.0)	12 (2.3)	52 (10.1)	95 (18.5)		
31–40	8 (1.6)	6 (1.2)	41 (8.0)	55 (10.7)		
41–50	8 (1.6)	5 (1.0)	41 (8.0)	54 (10.5)		
51–60	0 (0)	11 (2.1)	65 (18.1)	76 (14.8)		
61–73	6 (1.2)	38 (7.4)	153 (29.8)	197 (38.4)		
Ethnicity						
Malay	33 (6.4)	24 (4.7)	145 (28.3)	202 (39.4)	0.723	2.1
Chinese	33 (6.4)	37 (7.2)	162 (31.6)	232 (45.2)		
Indian	13 (2.5)	13 (2.5)	53 (10.3)	79 (15.4)		
Number of disabilities						
1 type of disability	26 (5.0)	35 (6.8)	155 (30.2)	216 (42.1)	0.169	6.4
2 types disabilities	22 (4.3)	21 (4.1)	80 (15.6)	123 (24.0)		
≥3 types of disabilities	31 (6.0)	18 (3.5)	125 (24.4)	174 (33.9)		
Total number of medical conditions						
None	12 (2.3)	5 (1.0)	28 (5.5)	45 (8.8)	0.010*	16.9
1 medical condition	39 (7.6)	25 (4.9)	119 (23.2)	183 (35.7)		
2 medical conditions	16 (3.1)	26 (5.1)	112 (21.8)	154 (30.0)		
≥3 medical conditions	12 (2.3)	18 (3.5)	101 (19.7)	131 (25.5)		
Patient complexity (BDA case-mix score)						
Standard complexity	3 (0.6)	4 (0.8)	26 (5.1)	33 (6.4)	0.381	8.6
Some complexity	26 (5.1)	25 (4.9)	101 (19.7)	152 (29.6)		
Moderate complexity	20 (3.9)	26 (5.1)	137 (26.7)	183 (35.7)		
Severe complexity	22 (4.3)	14 (2.7)	68 (13.3)	104 (20.3)		
Extreme complexity	8 (1.6)	5 (1.0)	28 (5.5)	41 (8.0)		

Table 3 shows the association between periodontal disease and demographic characteristics among the study population. Overall prevalence of unhealthy periodontium (BPE >0) was 418 (81.5%). Based on the bivariate analysis, the prevalence of periodontal disease was significantly associated with gender, age, number of disabilities, medical conditions, and BDA case mix complexity score. Males exhibited a higher prevalence of periodontitis (n = 123, 24.0%) than their female counterparts (p = 0.048). The prevalence of periodontal disease was significantly higher among patients aged 61 and older (p<0.001) compared to other age groups. In terms of ethnicity, Chinese patients had a higher prevalence of gingivitis (n = 74, 14.4%) and periodontitis (n = 125, 24.4%) than other ethnic groups; however, the difference was not statistically significant. Patients with three or more disabilities had a higher prevalence of periodontitis (n = 140, 27.3%) compared to those with one or two types of disabilities. A higher number of medical conditions was significantly associated with an increased prevalence of periodontal disease (p<0.001). Patients in the moderate (n = 183, 35.7%), some (n = 152, 29.6%), severe (n = 104, 20.3%), and extreme (n = 41, 8.0%) complexity categories had higher levels of periodontal disease compared to those in the standard complexity category.

**Table 3:** The association between periodontal disease at the baseline appointment and patients’ demographic characteristics (n = 513) ^a^Chi square test. ^*^The level of significance for this analysis was set at 0.05 BPE: Basic Periodontal Examination; BDA: British Dental Association

Variables	Frequency, n (%)					
	Healthy: BPE 0	Gingivitis: BPE 1-2	Periodontitis: BPE 3-4	Total	P-value^a^	Chi-square value
Gender						
Male	61 (11.9)	87 (17.0)	123 (24.0)	271 (52.8)	0.048*	6.1
Female	34 (6.6)	87 (17.0)	121 (23.6)	242 (47.2)		
Age groups, years						
16–20	12 (2.3)	14 (2.7)	10 (1.9)	36 (7.0)	<0.001*	117.6
21–30	42 (8.2)	33 (6.4)	20 (3.9)	95 (18.5)		
31–40	19 (3.7)	19 (3.7)	17 (3.3)	55 (10.7)		
41–50	11 (2.1)	26 (5.1)	17 (3.3)	54 (10.5)		
51–60	6 (1.2)	32 (6.2)	38 (7.4)	76 (14.8)		
61-73	5 (1.0)	50 (9.7)	142 (27.7)	197 (38.4)		
Ethnicity						
Malay	45 (8.8)	71 (13.8)	86 (16.8)	202 (39.4)	0.080	8.3
Chinese	33 (6.4)	74 (14.4)	125 (24.4)	232 (45.2)		
Indian	17 (3.3)	29 (5.7)	33 (6.4)	79 (15.4)		
Number of disabilities						
1 type of disability	55 (10.7)	92 (17.9)	69 (13.5)	216 (42.1)	<0.001*	118.4
2 types of disabilities	35 (6.8)	53 (10.3)	35 (6.8)	123 (24.0)		
≥3 types of disabilities	5 (5.3)	29 (5.7)	140 (27.3)	174 (33.9)		
Total number of medical conditions						
None	24 (4.7)	15 (2.9)	6 (1.2)	45 (8.8)	<0.001*	82.8
1 medical condition	47 (9.2)	72 (14.0)	64 (12.5)	183 (35.7)		
2 medical conditions	17 (3.3)	43 (8.4)	94 (18.3)	154 (30.0)		
≥3 medical conditions	7 (1.40)	44 (8.6)	80 (15.6)	131 (25.5)		
Patient complexity (BDA case-mix score)						
Standard complexity	7 (1.4)	10 (1.9)	16 (3.1)	33 (6.4)	0.032*	16.9
Some complexity	40 (7.8)	56 (10.9)	56 (10.9)	152 (29.6)		
Moderate complexity	27 (5.3)	65 (12.7)	91 (17.7)	183 (35.7)		
Severe complexity	15 (2.9)	34 (6.6)	55 (10.7)	104 (20.3)		
Extreme complexity	6 (1.2)	9 (1.8)	26 (5.1)	41 (8.0)		

A binary logistic regression analysis was performed to assess the association between selected patient demographic characteristics and the presence or absence of periodontal pockets (BPE ≥3) and cavitated caries experience (D3-6MFT >0) (Table 4). For this analysis, age was re-categorized into two groups (16-60 vs. 61-73 years) to improve model stability. Age group, ethnicity, and number of disabilities remained statistically significant predictors of periodontal pockets. Malays had significantly higher odds (AOR = 1.86; 95% CI: 1.10-3.15) of having periodontal pockets compared to Indians (reference group). Those aged 16-60 years had significantly higher odds of having BPE ≥3 (AOR = 5.42; 95% CI: 3.67-8.00) than those aged 61-73 years. Only the age group remained a significant predictor of dental caries experience in this study population. Patients aged 16-60 years had significantly higher odds of having caries experience compared to those aged 61-73 years (reference group), with an AOR of 9.56 (95% CI: 4.07-22.46).

**Table 4:** Binary logistic regression analysis for periodontal pocket BPE score ≥3 and dental caries experience D3-6MFT>0 among SHCN patients ^Binary logistic regression analysis. *The level of significance for this analysis was set at 0.05 Reference group: ^a^Gender: female. ^b^Ethnicity: Indian.^ c^Age group: 61-73 years. ^d^Patient complexity: extreme complexity. ^e^Number of disabilities: three or more disabilities. ^f^Number of medical conditions: none Periodontal disease model fit: Nagelkerke R² = 0.595 indicates that the model explained approximately 59.5% of the variance in the outcome. The model correctly classified 79.1% of cases. Dental caries model fit: Nagelkerke R² = 0.415 indicates that the model explained approximately 41.5% of the variance in the outcome. The model correctly classified 87.5% of cases. Variables were included using the enter method AOR: adjusted odd ratio; BPE: Basic Periodontal Examination; CI: confidence interval; DMFT: Decayed, Missing, or Filled Teeth; SHCN: special healthcare needs

Predictors	BPE score ≥3 (yes/no)				D_3-6_MFT >0 (yes/no)			
	AOR	95% CI		p-value^^^	AOR	95% CI		p-value^^^
		Lower	Upper			Lower	Upper	
Gender^a^								
Male	1.03	0.63	1.68	0.91	0.76	0.41	1.41	0.39
Ethnicity^b^								
Malay	1.86	1.10	3.15	0.02^*^	1.33	0.70	2.52	0.39
Chinese	1.18	0.56	2.48	0.66	1.25	0.53	2.94	0.61
Age group^c^, years								
16–60	5.42	3.67	8.00	<0.001^*^	9.56	4.07	22.46	<0.001^*^
Patient complexity^d^								
Standard complexity	0.70	0.27	1.87	0.48	0.33	0.07	1.55	0.16
Some complexity	0.67	0.25	1.79	0.43	0.67	0.14	3.15	0.61
Moderate complexity	0.48	0.16	1.43	0.19	0.59	0.12	2.86	0.52
Severe complexity	0.58	0.14	2.33	0.44	0.54	0.09	3.15	0.50
Number of disabilities^e^								
1 type of disability	0.92	0.50	1.71	0.80	0.71	0.32	1.59	0.41
2 types of disabilities	128.99	35.58	467.70	<0.001^*^	0.79	0.32	1.90	0.59
Number of medical conditions^f^								
1 medical condition	0.63	0.13	3.01	0.56	0.70	0.29	1.68	0.42
2 medical conditions	0.98	0.20	4.73	0.98	0.96	0.34	2.73	0.94
≥3 medical conditions	0.43	0.09	2.17	0.31	0.73	0.23	2.33	0.59

**Figure 1:**
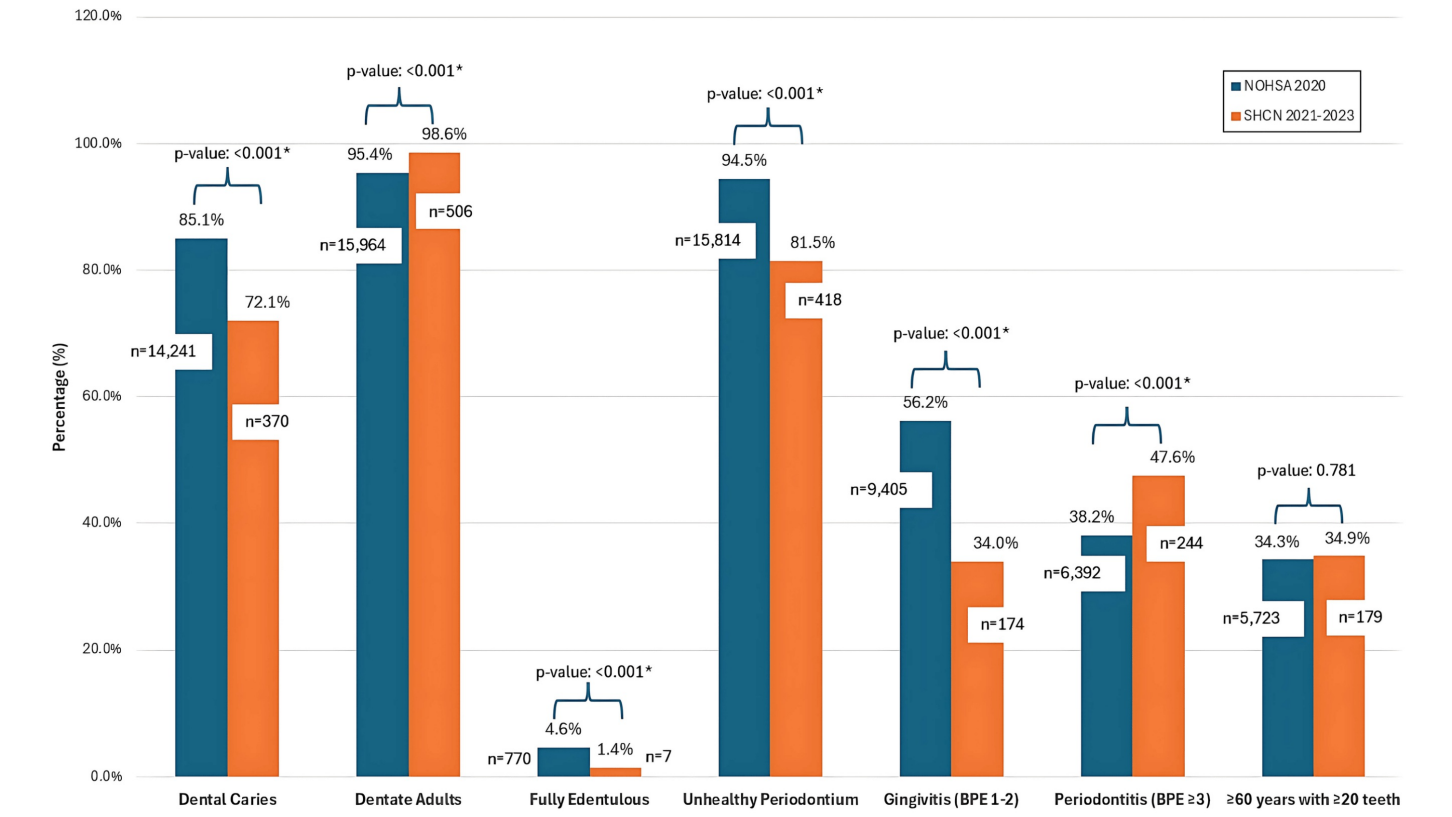
Comparison between data from the National Oral Health Survey of Adults-NOHSA (2020) and the study population (2021-2023) *The level of significance for this analysis was set at 0.05. Chi-square test was used to compare the data BPE: Basic Periodontal Examination; DMFT: Decayed, Missing, or Filled Teeth; NOHSA: National Oral Health Survey of Adults; SHCN: special healthcare needs

Data from the present study were compared with findings from the National Oral Health Survey of Adults 2020 in Malaysia [13] (Figure 1). The cavitated caries prevalence among the study population (SHCN) was significantly lower (n = 370, 72.1%) than the national prevalence (n = 14,241, 85.1%). However, the SHCN group had fewer remaining natural teeth (mean: 22.6) compared to general adult population (mean: 24.4). In terms of periodontal status, the prevalence of unhealthy periodontium (BPE >0) was higher in the general adult population (n = 15,814, 94.5%) than in the SHCN group (n = 418, 81.5%). However, the prevalence of periodontal pockets (BPE ≥3) was notably higher among SHCN patients (n = 244, 47.6%) compared to the general population (n = 6,392, 39.4%). The proposed null hypothesis, that there was no significant difference in oral health status between adult SHCN patients at a Malaysian dental hospital and the general adult population based on national data, was rejected.

## Discussion

This study aimed to evaluate the oral health status of adult patients with special healthcare needs (SHCN) at a Malaysian dental hospital, identify factors associated with their oral health outcomes, and compare their disease burden with national prevalence data. The findings highlight a substantial burden of dental caries and periodontal disease among adults with SHCN in Malaysia. The study's null hypothesis - that there was no significant difference in oral health status between Malaysian adult SHCN patients and the general adult population - was rejected. SHCN patients demonstrated a higher burden of periodontal disease and lower overall tooth retention compared to the national average. Poor oral health status in this study population was significantly associated with older age, multiple disabilities, and higher levels of clinical complexity. These findings highlight persistent oral health inequalities within Malaysia’s healthcare system, despite the availability of subsidized dental care through public services.

More than half of the study population were aged 50 years and above, with the majority classified as medically complex individuals living with disabilities. This finding aligns with studies from Australia, which also reported that a large proportion of individuals with SHCN (81.7%) were older adults with medical complexities [14-15]. The observed pattern underscores a broader demographic shift toward aging populations with multiple comorbidities, a trend also seen in Malaysia that significantly challenges the planning and delivery of comprehensive oral healthcare services [9]. Many of these patients suffer from conditions that compromise their ability to maintain oral hygiene, leading to an increased risk of dental problems. A study by Villa et al. (2015) demonstrated that adults with medical complexity had significantly higher risks of dental caries and oral infections due to contributing factors such as neuromuscular dysfunction, gastroesophageal reflux, polypharmacy, and medication-induced xerostomia [16-17].

In this study, most patients were classified as having moderate to severe complexity based on the BDA case mix [10], typically requiring a combination of restorative, periodontal, and preventive care. Managing such cases demands a dental workforce trained in specialized procedures, including behavior management, sedation, and general anesthesia [17]. Although general dentists may be able to provide basic care for some individuals with SHCN, many practitioners report a lack of confidence in treating SHCN patients, and hence tend to refer such cases for specialist care [18-19]. It was reported that the number of adult patients with SHCN attending SCD specialist clinics in Malaysia increased by 36.4% in the post-pandemic period from 2021 to 2022 [8]. This number is expected to continue rising due to the possibility of underreporting. Given the rising demand for complex care for adult patients with SHCN, it is essential to integrate early exposure into the undergraduate dental curriculum. This would help future practitioners develop the competence, confidence, and empathy needed to deliver inclusive care to this vulnerable population [20-22].

A high prevalence of dental caries and periodontal disease was observed in this study population, highlighting substantial unmet oral health needs among adults with SHCN. These findings align with reports from other countries such as India, Japan, the United States, and the United Kingdom [22-23], where SHCN populations also experience a disproportionate burden of oral disease. Several factors may contribute to this elevated disease burden, including socioeconomic status, hormonal changes, cultural dietary preferences (such as frequent consumption of cariogenic foods or beverages), and limited utilization of preventive oral care services [24].

Although the overall burden of dental caries and periodontal disease in the SHCN population was generally comparable to national averages, notable differences in disease patterns were observed. For example, individuals with SHCN in this study retained fewer natural teeth compared to the general adult population in Malaysia [13]. This may be attributed to the fact that the study population comprised patients who were already receiving care or had been referred for further management at a tertiary centre. It may also reflect a greater reliance on tooth extractions as a primary treatment approach, particularly in response to the high prevalence of advanced-stage oral disease among individuals with SHCN. Moreover, SHCN patients often seek dental care only when symptoms arise, leading to delayed treatment and more severe disease at the time of presentation [15,23].

Furthermore, this study found that periodontitis prevalence peaked in older adults and those with multiple disabilities and medical conditions. Similar findings have been reported in studies from India [25] and Japan [26], where elderly adults showed progressive attachment loss over the years. This suggests that individuals with complex health and functional limitations may be at greater risk of severe periodontal disease, which may lead to tooth loss. The significant association between periodontal pockets and the number of disabilities in the final regression model highlights the progressive nature of untreated gingival inflammation. Ageing, poor oral hygiene, and systemic inflammation further accelerate periodontal disease progression [5,27]. Given that SHCN patients often have underlying medical conditions, delayed dental care can worsen systemic health issues and complications related to poor nutrition due to difficulties in chewing and swallowing [28-29]. Therefore, implementing multidisciplinary collaboration, early detection, and caregiver-assisted oral hygiene routines is crucial in mitigating the impact of periodontal disease in individuals with SHCN [18].

In the bivariate analysis, older adults exhibited a higher prevalence of dentine caries and periodontal disease. However, the multivariate analysis revealed that adults aged 16-60 years had higher odds of developing dentine caries and periodontal disease compared to elderly aged 61-73 years. This finding should be interpreted with caution, as it may be influenced by the age group categorization in the regression model, where young and middle-aged adults were grouped together under the 16-60 category. A possible explanation for this finding is that the individuals in this age range are more likely to retain a greater number of teeth, thereby increasing their susceptibility to caries compared to older adults, who typically have fewer teeth [30]. A similar explanation may apply to the higher caries prevalence observed among the general adult population in Malaysia. This could be attributed to the sampling approach in this hospital-based study, which differs from the national survey [13].

The findings call for the strengthening of oral health policy for persons with disabilities in Malaysia, including expanding resources for SCD specialist clinics, integrating oral health into broader non-communicable disease and ageing strategies, and enhancing training for dental professionals. Moreover, investment in community-based dental outreach services, tele-dentistry, and mobile clinics is recommended to reduce physical and logistical barriers for adult patients with SHCN.

Study strengths and limitations

This study has several strengths. The use of clinical data enabled the inclusion of medically complex individuals and those with disabilities, an often underrepresented population in oral health research. The large sample provided valuable insights into oral health status, service utilization, and allowed comparison with national data. However, the findings should be interpreted in light of several limitations. Firstly, the retrospective study design relies on existing clinical records, which may be subject to missing data or incomplete documentation. Secondly, clinical assessments were performed by multiple clinicians, potentially introducing variability in clinical judgment. Nonetheless, all clinicians underwent regular calibration exercises at the hospital. Thirdly, the study was conducted at a single tertiary dental hospital, which may limit the generalizability of the findings to other regions in Malaysia. Finally, comparisons with national data may be challenged by differences in sampling procedures and the indices used to measure caries and periodontal disease. However, efforts were made to harmonize the outcome measures by adjusting the cut-off points used in the hospital data indices to align as closely as possible with those in the national survey. This adjustment allowed for a more meaningful comparison of oral disease patterns between the study population and the general adult population.

## Conclusions

This study highlights the high burden of dental caries and periodontal disease among adults with SHCN in Malaysia. However, the overall dental disease burden in this population is not substantially higher than the national average, except for the prevalence of periodontal pockets and the average number of teeth retained. Age was a significant predictor of dental caries, while both age and the number of disabilities were associated with periodontal disease. These findings emphasize the critical need for targeted preventive strategies and improved accessibility to oral healthcare services for this vulnerable population. Additionally, the results provide valuable insights to guide the expansion of SCD and community-based oral health services in Malaysia to better serve this group. Future research should involve larger, multi-center samples to enhance generalizability and include qualitative studies on patient and caregiver experiences in accessing dental care.
